# Are We Growing Too Fast?

**Published:** 1900-10

**Authors:** 


### ARE WE GROWING TOO FAST?

A WELL-BALANCED ration is the problem to-day in successful animal husbandry. It is the topic of every experiment station, agricultural laboratory, among practical breeders, stock-breeders' associations, and agricultural and stock-breeding journals of the world, havng for its basis the obtaining of the most for the least cost. It aims to secure energy, force, weight values as quickly as is consistent with safety.

May we not liken our Association in its remarkable growth to this great problem?

An organization that is banded together for scientific progress, which has changed its total membership twice in ten years, its field of growth to newer territorial pastures, is confronted with a serious problem in assimilation economics as to how best it can balance the growth of so great an organization and expend well its energy, direct efficiently its force, maintain its resources safely, and reap as its harvest a well-balanced progress along the line of Association work and usefulness.

The struggle for a large Association numerically has gone on apace, but how well we assimilate the added energy, force, power, and worth of the yearly harvest is a matter for serious reflection. A year or two of thoughtful planning as to how best we can assimilate the great wealth of numbers, to make them more useful and efficient in true Association advancement, is a serious problem of the hour.

